# Impact of pasireotide on postoperative pancreatic fistulas following distal resections

**DOI:** 10.1007/s00423-021-02083-2

**Published:** 2021-01-20

**Authors:** Tiina Vuorela, H. Mustonen, A. Kokkola, C. Haglund, H. Seppanen

**Affiliations:** 1 Department of Surgery, Helsinki University Hospital, University of Helsinki, Helsinki, Finland; 2 Translational Cancer Medicine Research Program, Faculty of Medicine, Helsinki University Hospital, University of Helsinki, Helsinki, Finland

**Keywords:** Pancreatic surgery, Pancreatic distal resection, Pancreatic fistula, Postoperative complications

## Abstract

**Purpose:**

Postoperative pancreatic fistula (POPF), a difficult complication after surgery, can cause peripancreatic fluid collection and infections in the operative area. In addition, pancreatic fluid is corrosive and can lead to postoperative bleeding. Clinically significant grade B and C fistulas (CR-POPF) increase postoperative morbidity, resulting in a prolonged hospital stay. Delaying adjuvant therapy due to fistula formation in cancer patients can affect their prognosis. In this study, we aimed to determine if pasireotide affects fistula formation, and the severity of other complications in patients following pancreatic distal resections.

**Data and methods:**

Between 2000 and 2016, 258 distal pancreatectomies were performed at Helsinki University Hospital and were included in our analysis. Pasireotide was administered to patients undergoing distal resections between July 2014 and December 2016. Patients received 900-μg pasireotide administered twice daily perioperatively. Other patients who received octreotide treatment were analyzed separately. Complications such as fistulas (POPF), delayed gastric emptying (DGE), postpancreatectomy hemorrhage (PPH), reoperations, and mortality were recorded and analyzed 90 days postoperatively.

**Results:**

Overall, 47 (18%) patients received pasireotide and 31 (12%) octreotide, while 180 patients (70%) who received neither constituted the control group. There were 40 (16%) clinically relevant grade B and C POPFs: seven (15%) in the pasireotide group, three (10%) in the octreotide group, and 30 (17%) in the control group (*p* = 0.739). Severe complications categorized as Clavien–Dindo grade III or IV were recorded in 64 (25%) patients: 17 (27%) in the pasireotide group, 4 (6%) in the octreotide group, and 43 (67%) in the control group (*p* = 0.059). We found no 90-day mortality.

**Conclusions:**

In this study, pasireotide did not reduce clinically relevant POPFs or severe complications following pancreatic distal resection.

## Introduction

Complications following pancreatic surgery are potentially life threatening and influence patients’ quality of life. Morbidity following pancreatic surgery varies depending on the operating center and the extent of the surgery. Some type of complication occurs in roughly 30% to 50% of patients [1]. Following distal resections, one of the most severe complications is a pancreatic fistula (POPF). According to a 2014 systematic review by Harnoss et al., following the consensus statement criteria, the median rate of clinically relevant POPF (CR-POPF) was 17% [2]. Other potentially harmful complications following pancreatic distal resections include delayed gastric emptying (DGE), postpancreatectomy hemorrhage (PPH), and infections in the operative area [3].

POPF can form in the resection area or elsewhere in the remaining pancreas. Amylase-rich pancreatic fluid is corrosive and may lead to life-threatening hemorrhaging postoperatively. In addition, infections and abscesses can form due to fluid leakage from the pancreatic tissue or anastomosis. POPF diagnosis is determined based on criteria outlined by an international study group (ISGPS), typically indicated when the drain fluid amylase concentration is three times the normal serum amylase level combined with typical clinical manifestations of a fistula [4]. Grade B and C fistulas are distinguished based on the severity of the clinical manifestations, and the surgical procedures performed. Furthermore, CR-POPF patients require specific treatment, such as repeated imaging, parenteral nutrition, antibiotics, somatostatin analogs, or persistent drainage for more than 3 weeks. Moreover, patients with a grade C fistula exhibit clear signs of infection, sepsis, and the need for re-operation. Mortality in this group can reach 35% [5].

Fistulas carry a rather specific importance to both patients and institutions. They may affect patient survival if adjuvant therapy is delayed following surgery for cancer [6]. In addition, a prolonged hospital stay and necessary follow-up procedures also carry economic consequences for hospitals.

Somatostatin and its analogs have served as the primary course of treatment for pancreatic fistulas for years, although previous data seem somewhat inconsistent. For instance, in 2000, Yeo and colleagues carried out a randomized trial with a somatostatin analog, octreotide, administered preoperatively to pancreaticoduodenectomy patients, finding no positive effect on the fistula rate or other postoperative complications [7]. By contrast, Allen and associates completed a randomized prospective trial using pasireotide, a somatostatin analog with a longer half-life than octreotide that binds to four out of five somatostatin receptor subtypes. Allen et al. described a significant decrease in clinically relevant POPFs in 80 patients undergoing distal pancreatic resection, ranging from 23% in the placebo group to 7% in the pasireotide group [8].

Here, we attempted to validate the finding that pasireotide administration associates with the fistula rate or other postoperative complications following distal pancreatic resections.

## Patients and methods

In total, between 2000 and 2016 at Helsinki University Hospital, 276 elective distal pancreatectomies were performed. In this retrospective study, we included in our analysis 258 of those distal resections. Pasireotide was administered preoperatively to all patients undergoing distal pancreatic resection between July 2014 and December 2016. Prior to 2014, octreotide administration was decided upon at the operating surgeon’s pre- or intraoperatively discretion. All other 180 (70%) distal resection patients operated on between 2000 and 2016 who did not receive somatostatin analogs constituted the control group. A total of 17 patients were identified as not receiving pasireotide between 2014 and 2016 at the operating surgeon’s discretion; these patients were analyzed as a part of the control group. We excluded 18 distal resection patients from the study; one patient received both pasireotide and octreotide, for whom drug delivery was stopped after 1 day, and was thus excluded from the analysis. We also excluded ten patients for whom data were missing. Seven patients were excluded because they were recruited to another clinical study.

In total, 47 of 258 patients (18%) received 900-μg pasireotide administered subcutaneously twice daily, beginning on the morning of the surgical day, and continuing until discharge from the hospital, for a maximum of 7 days postoperatively. We analyzed separately 31 (12%) patients who received octreotide perioperatively. Octreotide was administrated through a 200-μg infusion twice daily for 3 days beginning on the surgical day (see Table 1).

**Table 1:** Patient demographic characteristics

Characteristics				
Baseline (*n* = 258)	Pasireotide (*n* = 47)	Octreotide (*n* = 31)	Control (*n* = 180)	*P* value
Female (%)	26 (55)	21 (68)	104 (58)	
Male (%)	21 (45)	10 (32)	76 (42)	0.554
Age, in years (range)	65 (18–82)	58 (23–77)	62 (19–84)	0.454
Operation				0.000
Open (%)	26 (55)	29 (94)	147 (82)	
Minimally invasive surgery (%)	21 (45)	2 (6)	33 (18)	
Diagnosis (%)				0.000
Pancreatic adenocarcinoma	9 (19)	0	41 (23)	
Chronic pancreatitis	0	3 (10)	20 (11)	
Mucinous cystic neoplasia	3 (6)	4 (13)	15 (8)	
Serous cystic neoplasia	2 (4)	1 (3)	13 (7)	
Neuroendocrine neoplasia	19 (40)	15 (48)	38 (21)	
IPMN	5 (10)	0	23 (13)	
Other^a^	10 (21)	7 (23)	30 (17)	
Postoperative				
Hospital stay, in days (range)^b^	9 (6–13)	8 (5–39)	8 (3–65)	0.834
Drain removal, pod (range)	4 (0–35)	4 (1–16)	4 (0–39)	0.210
Readmission (%)	10 (22)	3 (10)	30 (17)	0.401
Somatostatin analog administration median, in days (range)	7 (1–19)	5 (1–7)	0	

*P* values are calculated with either the Kruskal–Wallis test or with the Fisher-Freeman-Halton test.

^a^Other diagnoses (see text)

^b^Hospital stay in days does not include days after readmission

The drug used in this study, pasireotide (Signifor®), was supplied by Novartis Europharm Ltd. (Novartis, Basel, Switzerland). The drug manufacturer did not take part in the study nor did it cover any costs.

We collected information about patient age, gender, the American Society of Anesthesiologists (ASA) Physical Status Classification, tumor histology, grade, and the type of resection. In addition, we recorded postoperative information, the length of the hospital stay, fever following surgery (> 38 °C), complications, and surgical, and other re-operations.

Patient diagnoses were heterogeneous. In both groups, the majority of diagnoses consisted of neuroendocrine tumors of all grades. Other diagnoses consisted of adenocarcinoma, metastatic carcinomas (renal, colon, and gastric metastases), and lymphomas. Table 1 summarizes the patient demographic characteristics. The analysis of complications was based on POPF, DGE, and PPH, according to international consensus statements (i.e., ISGPS) [3, 4, 9]. Complications were classified during a 90-day follow-up period according to the Clavien–Dindo classification [10, 11]. Postpancreatectomy fistula diagnosis was determined if on the third postoperative day (POD), the drain amylase level was three times higher than the upper normal blood amylase limit (dr-amyl, IU/ml). Drain removal was decided upon after ensuring normal amylase levels or if the drain output ceased. Patient discharge from the hospital was planned after peroral nutrition, mobilization, and postoperative pain was managed without requiring outside assistance, and the patient displayed no signs of complications. In the case of CR-POPF, the patient could be discharged from the hospital with a drain. Removal of the drain was performed in the outpatient clinic within 7 to 14 days, or a step-by-step retraction of the drain was performed permitting the operating area to heal properly.

The annual volume of distal pancreatic resections increased during our study period, reaching about 20 to 30 procedures per year from 2014 to 2016. In total, five pancreatic surgeons, including one who retired, operated on the patients included in this study. The majority of patients (*n* = 202; 78%) were operated on through a laparotomy and 56 (22%) through minimally invasive techniques. In the laparotomy, the pancreatic resection line was closed either using sutures or with a stapler. In the minimally invasive procedures, stump closure was carried out with a stapler. There is no record of which staplers were used prior to 2012. Postoperatively, one drain was left close to the resection line in all patients.

Data were analyzed using IBM’s SPSS version 22, using the Fisher-Freeman-Halton exact tests or the Kruskal–Wallis test. Cramer’s V with bootstrapped 95% (1000 samples) confidence intervals (CIs) was calculated for RxC tables to evaluate the effect size of the association between variables. Multivariate logistic regression was performed in the adjusted analysis. We considered *P* < 0.05 as statistically significant using two-tailed tests.

This retrospective study relied on data collected from patient records at the Helsinki University Hospital. The study was approved by the Surgical Department, and the Surgical Ethics Committee of Helsinki University Hospital (Dnro HUS 226/E6/06, extension TMK02 §66 17 April 2013) and was carried out in accordance with the Helsinki Declaration. We report our findings in accordance with the STROCSS criteria [12].

## Results

Patients receiving pasireotide postoperatively stayed in the hospital for a median of 9 days (range, 6-13 days), while the median hospital stay in the octreotide group and control group was 8 days (range 5–39 days vs. 3–65 days, respectively). The drain was removed in the pasireotide, and octreotide group on median day 4 (ranges, 0-35 vs. 1–16 days, respectively). The hospital readmission rate was higher in the pasireotide group than in the octreotide or control group (22% vs. 10% and 17%, respectively). Table 1 summarizes the patient demographic characteristics.

The number of clinically relevant grade B and C POPFs was 40 (16%; *p* = 0.739). CR-POPF in the pasireotide group reached 15% compared with 10% in the octreotide group and 17% in the control group (*p* = 0.630). We found no statistically significant difference between groups (Table 2). In addition, patients with an open procedure had 32 (16%) compared with 7 (12%) CR-POPFs in the minimally invasive group (*p* = 0.348). Table 8 in the appendix shows the adjusted logistic regression analysis for the open procedure compared with the minimally invasive technique and pathological diagnoses (p = 0.841; Appendix, Table 5), revealing no significant difference between the pasireotide or octreotide group compared with the control group. Other risk factors, such as the gland structure (soft vs. hard) and the pancreatic duct diameter, were not systematically recorded throughout our study.

**Table 2:** Post-pancreatectomy fistulas (POPF) based on severity

POPF	No fistula	BL (%)	*B* (%)	*C* (%)	*P* value
Pasireotide (*n* = 48)	29 (60)	12 (25)	7 (15)	0	
Octreotide (*n* = 31)	23 (74)	5 (16)	2 (7)	1 (3)	
Control (*n* = 180)	115 (64)	37 (21)	26 (14)	2 (1)	0.630

*BL* biochemical leak, *B/C* clinically relevant post-pancreatectomy fistula (CR-POPF)

*P* value for the control row is for the entire table, octreotide and pasireotide compared with the control group. Cramer’s V for clinically relevant fistula (*B*/*C*) vs. no fistula or BL is 0.062 (95% confidence interval 0.020–0.177, *p* = 0.615)

In addition, we identified no 90-day mortality. Overall, 25% of patients in this study had a Clavien–Dindo classification of III to IV complications: 36% in the pasireotide group compared with 13% in the octreotide group and 24% in the control group. In addition, more patients from the pasireotide group were readmitted to hospital (21% vs. 10% and 17%). These differences were not, however, statistically significant (Table 3). Furthermore, we found that Clavien–Dindo grade III to IV complications occurred in 44 patients (29%) undergoing an open procedure compared with nine patients (19%) in the minimally invasive group (*p* = 0.080). Among all Clavien–Dindo grade III to IV complications, 50 patients (80%) had surgical, and 47 patients (76%) had a nonsurgical complication. More specifically, among the surgical complications, we identified surgical site infection in 12 patients (4%), a chyle leak in two patients (1%), and an endoscopic procedure performed on 24 CR-POPF patients (9%). In addition, 43 patients (17%) were readmitted to hospital during the 90-day follow-up: seven had an infection in the surgical area which included one POPF A, for which only antibiotic treatment was administered; 22 readmitted patients had a POPF B requiring an ultrasound-guided drainage or endoscopic procedure combined with antibiotic treatment if an infection was detected; and 4 patients had a POPF grade C. Among the patients readmitted, one patient had a chyloascites drained, two patients had a wound infection, three had a pneumonia, two patients were admitted with ileus, which resolved through a conservative care, one patient had a pulmonary embolism, and one patient was admitted for inadequate pain medication. In our analysis, we detected a need for re-operation in nine (3%) cases; post-pancreatectomy hemorrhage, perforated intestine, necrotic spleen, or two grade C fistulas needed a laparotomy. Among nonsurgical complications, we identified a thromboembolic complication in nine patients (3%), pneumonia in 26 patients (10%), pleural effusion in 21 patients (8%), cardiopulmonary complications in 8 patients (3%), ileus in 4 patients (2%), clostridium difficile-diarrhea in 4 patients (2%), urinary tract infections in 6 patients (2%), and neurological complications in 5 patients (2%). We detected no differences in postoperative complications between a minimally invasive surgery and open distal resections. In addition, fistulas stemming from different procedures appear in the appendix in Table 4. Postoperative drains were left in all patients except one undergoing a minimally invasive surgery, for whom the operating surgeon decided to leave out the drain.

**Table 3:** Complications according to the Clavien-Dindo score of 0 to 5

Clavien–Dindo	0 (%)	1 (%)	2 (%)	3 (%)	4 (%)	5 (%)	*P* value
Pasireotide (*n* = 47)	2 (4)	13 (28)	15 (32)	16 (34)	1 (2)	0	0.097
Octreotide (*n* = 31)	4 (13)	14 (45)	9 (29)	4 (13)	0 (0)	0	0.243
Control (*n* = 180)	34 (19)	51 (28)	51 (28)	40 (22)	3 (2)	0	0.195

Clavien–Dindo (CD) values were grouped for comparisons (CD3–5 vs. CD0–2), and *P* values were obtained using the Fisher’s exact test. The *P* value for the control row is for the entire table, and others are compared with the control. Cramer’s V is 0.149 (95% confidence interval 0.060–0.289, *p* = 0.063). Other: lymphoma, benign cyst, metastasis of renal, colon, adrenal, gastric, seminal and ovarian carcinomas, excess spleen, pseudopapillary neoplasia, sarcoma, paraganglioma, osteoclast giant-cell tumor

*MIS* minimally invasive surgery, *IPMN* intraductal pancreatic mucinous neoplasm, *d* day, *pod* postoperative day, *yrs* years, *POPF* post-pancreatectomy fistula, *BL* biochemical leak

In our study, grade B and C PPHs occurred in 38 patients (15%). Two patients with grade B POPFs underwent a re-operation due to an early severe PPH, and three patients with a grade C late PPH required a repeat laparotomy and one angioembolization. We found no statistically significant difference in PPH between the pasireotide, octreotide, and control groups (*p* = 0.633; Appendix, Table 6).

Across all patients, a grade A DGE was observed in 11 (4%), grade B in three (1%), and grade C in one patient. Neither the pasireotide nor the octreotide group had a grade B or C DGE. No significant difference was detected between the groups (*p* = 0.447; Appendix, Table 7).

## Discussion

We studied the effect of perioperative pasireotide in all patients operated on in our hospital who underwent pancreatic distal resection, finding no reduction in postoperative fistula formation or other complications. The use of pasireotide in high-risk patients at Helsinki University Hospital was initiated following Allen’s 2014 randomized study, which reported that pasireotide halved the frequency of CR-POPFs following distal pancreatic resection [8]. Pancreatic distal resection is considered a high-risk procedure for fistula. Before 2014, surgeons used octreotide, switching to pasireotide following Allen’s study. There were no specific study criteria other than the surgeon’s preference determining the choice of medication. Subsequent to Allen’s study, other reports on the effect of pasireotide appeared. Because of conflicting results, however, no consensus exists on whether pasireotide prophylaxis is warranted considering its high cost. Allen’s study group published results after routine pasireotide use in 2019, a result suggesting a similar positive effect to that from the first randomized study, although no statistical difference in the distal pancreatectomy group was found [13]. At our institution, we studied the effect of pasireotide in high-risk patients after pancreaticoduodenectomy, demonstrating the prophylactic effect on fistula formation [14]. In 2017, a German study group published their results and found no effect on the fistula rate among any somatostatin analogs [15]. Recently, two other studies found that pasireotide carried no effect on fistula formation. In Elliot’s prospective study, pasireotide had no prophylactic effect on fistula formation in distal pancreatic resections [16]. In addition, Young et al. in their prospective trial found no effect of pasireotide on fistula formation following distal resections. Overall, they found a high fistula risk of 28.4% [17].

Previously, octreotide has been the most commonly used prophylactic agent to prevent pancreatic fistulas. A 2017 systematic review and meta-analysis by Ma et al. proposed that octreotide could prove effective not just in preventing fistula formation, but also in preventing fluid collection and postoperative pancreatitis [18]. A reduction in postoperative fistulas following perioperative octreotide was also described by Ridolfini et al. in 2007 [19]. Before Allen’s 2014 report, pasireotide and its effects in pancreatic surgery were unknown, and octreotide was used in our hospital at the surgeons’ discretion, perhaps representing a bias in our study population. Furthermore, because multiple patients received octreotide postoperatively in our study cohort, we sought to analyze the octreotide group separately in order to minimize any confounding factors compared with the no-medication group. The common use of octreotide and pasireotide remains controversial, and their use continues to differ between institutions and operating surgeons.

Laparoscopic distal resections gained popularity at our institution in 2011, perhaps explaining the differences in the number of procedures between the pasireotide and control groups. There was no record of which staplers were used in our data, while no studies concerning different staplers affecting fistula formation have emerged. Several studies attempted to examine post-pancreatectomy fistulas by comparing different stump-closure techniques; none has succeeded in noting differing results [20–22]. A consensus statement on diagnosing pancreatic fistula appeared in 2006, and the updated statement published in 2016, which we used in this study [1, 4]. Studies carried out before then might have applied different diagnostic criteria, while varying practices concerning drain replacement, and removal following surgery continue to characterize individual studies.

Patient selection spanned a long period of time, possibly affecting the outcomes in our study. Moreover, as seen in many retrospective studies, some important data might be missing due to the reliance on older patient records. In recent years, in-hospital care has focused on enhancing patient recovery. Postoperative hospital stays have declined, although this appears not to have influenced complications [23]. One limitation to our study includes our reliance on retrospective data from one institution. Despite being a retrospective study, our results add up to the number of patients in other studies, studies which identified no reduction in postoperative complications through perioperative pasireotide use.

Overall, post-pancreatectomy hemorrhage was diagnosed using the ISGPS consensus statement, with an incidence of 15% in our patient population, a figure which is quite high. Most patients who received blood products were part of the early 2000s control group, and the limits for blood transfusions were 100 g/l. Since then, the limits have decreased to more conservative values depending on the patient’s co-morbidities and clinical status. DGE remained rare in our clinic, and we observed no effect from pasireotide. DGE rarely poses a problem in patients undergoing distal resection, but appears more common following pancreaticoduodenectomy [24, 25].

The diagnoses in the patients in our study were heterogeneous. There were many benign cysts and metastases from multiple different carcinomas, as shown in Table 1. While a previously published high-volume randomized controlled trial exists, the results remain unsubstantiated by other researchers. In the future, a multicenter randomized controlled trial would prove beneficial, specifically by comparing a placebo to pasireotide in pancreatic distal resections only. Perhaps this would clarify the benefit of using pasireotide in routine clinical practice.

In conclusion, in our study, pasireotide did not reduce CR-POPFs or other severe complications following pancreatic distal resection.

## Data Availability

Data and materials are not available for distribution because we do not have permission to share sensitive patient material.

## Appendix

**Table 4:** Postoperative pancreatic fistulas (POPF) between procedures

POPF	No fistula	BL (%)	*B* (%)	*C* (%)	*P* value
Open	125 (62)	43 (21)	29 (14)	4 (2)	
MIS	33 (58)	16 (28)	8 (14)	0 (0)	0.650

*MIS* minimally invasive surgery

**Table 5:** Pancreatic fistulas according to diagnoses

POPF	No fistula	BL (%)	*B* (%)	*C* (%)	*P* value
Adenocarcinoma	33 (66)	12 (24)	4 (8)	1 (2)	
IPMN	17 (61)	8 (29)	2 (7)	1 (4)	
Neuroendocrine neoplasia	39 (57)	16 (24)	12 (18)	1 (2)	
Chronic pancreatitis	16 (70)	2 (9)	4 (17)	1 (4)	
Mucinous cystic neoplasia	11 (48)	8 (35)	4 (17)	0	
Serous cystic neoplasia	12 (75)	2 (14)	2 (14)	0	
Other^a^	30 (60)	11 (18)	9 (15)	0	0.841

*POPF* postoperative pancreatic fistula, *BL* biochemical leak, *IPMN* intraductal mucinous pancreatic neoplasia

^a^Other: lymphoma, benign cyst, metastasis of renal, colon, adrenal, gastric, seminal and ovarian carcinomas, excess spleen, pseudopapillary neoplasia, sarcoma, paraganglioma, osteoclast giant-cell tumor, and hypernephroma

**Table 6:** Delayed gastric emptying (DGE), pasireotide and octreotide compared with the control group

DGE	None	*A* (%)	*B* (%)	*C* (%)	*P* value
Pasireotide	44 (17)	3 (1)	0	0	
Octreotide	30 (12)	1 (0.4)	0	0	
Control	169 (66)	7 (3)	3 (1)	1 (0.4)	0.447

**Table 7:** Post-pancreatectomy hemorrhage (PPH), pasireotide, and octreotide compared with the control group

PPH	None	*A* (%)	*B* (%)	*C* (%)	*P* value
Pasireotide	21 (8)	20 (8)	5 (2)	1 (0.4)	
Octreotide	15 (6)	13 (5)	3 (1)	0	
Control	73 (28)	77 (30)	27 (11)	3 (1)	0.221

**Table 8:** Logistic regression analysis of the risk of clinically relevant fistulas (CR-POPF) in distal pancreatectomy patients

		Univariate			Multivariate	
	OR	95% Cl	*P* value	OR	95% Cl	*P* value
Study arm						
Control	1			1		
Pasireotide	0.87	0.367–2.196	0.813	0.941	0.363–2.436	0.900
Octreotide	0.536	0.153–1.876	0.329	0.409	0.113–1.479	0.173
Procedure						
MIS vs. Open	0.727	0.303–1.745	0.476	0.581	0.224–1.506	0.264
Diagnosis						
Adenocarcinoma	1			1		
IPMN	1.214	0.301–4.902	0.786	1.427	0.348–5.857	0.622
Neuroendocrine neoplasia	2.071	0.677–6.337	0.202	2.850	0.880–9.230	0.081*
Chronic pancreatitis	2.444	0.630–9.481	0.196	2.631	0.631–0.670	0.166
MCN	1.320	0.287–6.071	0.721	1.572	0.337–7.335	0.565
SCN	1.257	0.219–7.210	0.797	1.523	0.258–8.971	0.642
Other^a^	1.932	0.598–6.243	0.271	2.264	0.688–7.445	0.179

Data are given as odds ratio (95% confidence interval). **P* < 0.1

*MIS* minimally invasive surgery, *IPMN* intraductal pancreatic mucinous neoplasia, *MCN* mucinous cystadenoma, *SCN* serous cystadenoma

^a^Other; lymphoma, benign cyst, metastasis of renal, colon, adrenal, gastric, seminal and ovarian carcinomas, excess spleen, pseudopapillary neoplasia, sarcoma, paraganglioma, and osteoclast giant-cell tumor
